# A Simple and Sensitive Wearable SERS Sensor Utilizing Plasmonic-Active Gold Nanostars

**DOI:** 10.1021/acsomega.4c05140

**Published:** 2024-09-05

**Authors:** Supriya Atta, Yuanhao Zhao, Sebastian Sanchez, Tuan Vo-Dinh

**Affiliations:** † Fitzpatrick Institute for Photonics, Duke University, Durham, North Carolina 27708, United States; ‡ Department of Biomedical Engineering, Duke University, Durham, North Carolina 27708, United States; § Department of Chemistry, Duke University, Durham, North Carolina 27708, United States

## Abstract

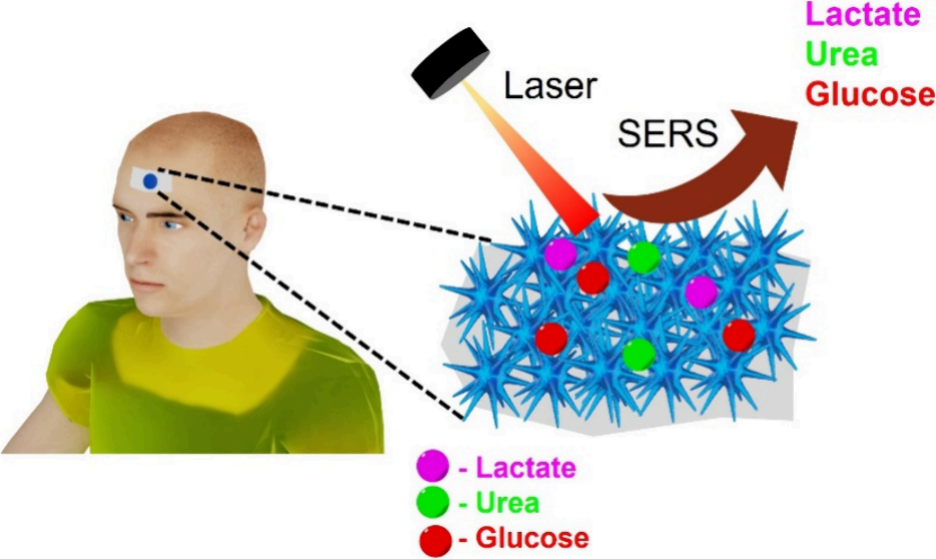

Wearable sweat sensors hold great potential for offering detailed health insights by monitoring various biomarkers present in sweat, such as glucose, lactate, uric acid, and urea, in real time. However, most previously reported sensors, primarily based on electrochemical technology, are limited to monitoring only a single analyte at a given time. This study introduces a simple, sensitive, wearable patch based on surface-enhanced Raman spectroscopy (SERS), integrated with highly plasmonically active sharp-branched gold nanostars (GNS) for the simultaneous detection of three sweat biomarkers: lactate, urea, and glucose. We have fabricated the GNS on commercially available adhesive tape, resulting in achieving a low-cost, flexible, and adhesive wearable SERS patch. The limits of detection for lactate, urea, and glucose were achieved at 0.7, 0.6, and 0.7 μM, respectively, which are significantly lower than the clinically relevant concentrations of these biomarkers in sweat. We further evaluated the performance of our wearable SERS patch during outdoor activities, including sitting, walking, and running. To evaluate its overall effectiveness, we simultaneously measured the concentrations of lactate, urea, and glucose during these activities. Overall, our simple, sensitive wearable SERS sensor represents a significant breakthrough by enabling the simultaneous detection of lactate, urea, and glucose present in sweat, marking a major step toward future applications in autonomous and noninvasive personalized healthcare monitoring at home.

## Introduction

With the rapid rise in public health concerns, capturing a comprehensive view of an individual’s physiological status at a deeper molecular level is essential, providing insights into glucose levels, metabolic waste products, and other vital small molecules present in our body.^1,2^ Various small-molecule biomarkers, such as lactate, glucose, urea, and uric acid, are present in body fluids and serve as important indicators of the body’s physiological state and health.^3−5^ For example, glucose concentration in body fluids is a crucial marker for evaluating glucose metabolism disorders, with high blood glucose levels typically associated with diabetes.^6^ Similarly, urea, a metabolic product found in body fluids, is a critical biomarker for renal function, and elevated urea levels in sweat are linked to an increased risk of heart failure.^7^ Traditionally, the concentration of these biomarkers is determined through invasive and often painful blood tests, which require a laboratory set up. With the rapid rise in public health concerns, there is a pressing need for medical screening that allows for monitoring biological molecules, providing a practical solution for on-site, real-time analysis without the need for complex laboratory equipment. Over the past two decades, remarkable advances in nanotechnology have driven groundbreaking innovations in wearable sweat sensor technology.^2,8−12^ These sensors are capable of real-time monitoring of critical biomarkers in easily accessible bodily fluids like sweat. They provide comprehensive insights into an individual’s physiological health, enabling noninvasive and real-time tracking of small biomarker molecules such as lactate, urea, glucose, and uric acid.^12−14^ Such advancements have led to more effective treatments and timely diagnoses, significantly enhancing the public healthcare system. Various wearable sensors have been developed, primarily based on electrochemical sensors.^1,15−17^ However, electrochemical sensors have limitations, including the inability to detect multiple biomarkers simultaneously and the high cost of substrate design.^18,19^ Therefore, there is a significant demand for the development of effective wearable sensors that offer cost-effective multiplex biomarker detection capabilities and are reusable, ultimately providing all populations with access to personalized healthcare.

Fifty years ago, Martin Fleischmann and co-workers observed in 1974 that the Raman scattering signal of pyridine adsorbed on a roughened silver electrode was significantly enhanced compared to the signal from pyridine in solution.^20^ Initially attributed to an increased surface area, the enhancement was later discovered in 1977 by Van Duyne and Creighton and their co-workers as an effect due to the interaction between the molecules and the metal surface, hence the name surface-enhanced Raman scattering (SERS).^21,22^ This discovery sparked significant interest in the fundamental study of this new SERS-based spectrochemical method over the next decade. However, the potential of SERS as an analytical tool diminished in subsequent years due to practical issues, particularly the difficulty in reproducibly preparing electrodes or colloidal metal nanoparticles, which were the main SERS platforms at the time. Additionally, the development of SERS as a general analytical method was limited because the SERS effect was only reported for a few highly polarizable small molecules, such as pyridine, benzoic acid, and their derivatives. Consequently, SERS found very few practical applications before the mid-1980s. In 1984, our laboratory reported the general applicability of SERS as an analytical technique, demonstrating that the SERS phenomenon is not limited to a few molecules but is a general effect that can be applied to a wide variety of chemicals, including homocyclic and heterocyclic polyaromatic compounds,^23^ The SERS method has emerged as an excellent analytical technique that provides unique spectral fingerprint information based on the specific structural vibrations of the target small molecule. SERS has been widely used for detecting small molecules across various fields, including food safety, biomedical sensing, early disease detection, environmental surveillance, homeland security, and more.^24−28^ Our laboratory has developed various plasmonic-active platforms for a wide range of SERS applications in chemical and biological sensing.^29,30^ The SERS effect results in an ultrahigh enhancement of weak Raman signals from analyte molecules, attributed to both the electromagnetic mechanism (EM) and the chemical mechanism (CM), with EM enhancement contributing the most. EM enhancements occur in plasmonic noble metal nanostructures, where a strong local electric field generated by laser excitation, known as localized surface plasmon resonance (LSPR), interacts with the analyte molecules. This interaction increases the polarizability of the molecules, significantly enhancing their Raman signals. It is well established that anisotropic nanoparticles generate intense localized electric fields at their sharp edges and tips, known as SERS “hot spots.” Consequently, much effort has been devoted to controlling the morphology of plasmonic noble metal nanoparticles to enhance the SERS signal. For instance, our laboratory was the first to introduce GNS as an SERS-enhancing platform.

Recently, the development of wearable SERS patches has attracted significant interest, driven by the demand for continuous, noninvasive monitoring in healthcare and security applications.^31,32^ Integrating SERS technology into wearable formats allows for the detection of trace amounts of substances, offering a practical solution for on-site, real-time analysis without the need for complex laboratory equipment.^33^ The development of wearable SERS patches involves various fabrication methods, each aiming to optimize the sensitivity, stability, and flexibility of the devices.^34^ Common approaches include depositing metallic nanostructures, such as gold or silver nanoparticles, onto flexible substrates. Techniques like inkjet printing, dip-coating, electrospinning, and nanoimprinting lithography have been extensively explored.^35−38^ For example, a highly scalable wearable SERS sensor using an ultrathin, flexible, and stretchable gold nanomesh was developed to detect sweat biomarkers such as urea.^32^ Recently, a wearable SERS sensor using inverted bimetallic nanopyramids (i-NPyr) on flexible plastic substrates, employing electron beam lithography and nanoimprinting lithography techniques, was introduced to detect urea and lactic acid in sweat.^39^ Flexible, nanoporous SERS substrates were designed to enrich and detect analytes in sweat.^40^

Despite the promising potential of wearable SERS patches, several disadvantages have been identified for their point-of-care application, such as the need for sensitive Raman instrumentation to achieve high sensitivity.^41,42^ Currently, there are no reports on the preparation of a simple, sensitive substrate capable of detecting multiple biomarkers, such as urea, lactic acid, uric acid, and creatinine, primarily due to the poor sensitivity of existing SERS substrates. Additionally, the cost and complexity of fabrication processes can hinder the scalability of wearable SERS patches. Advanced techniques like electron beam lithography and nanoimprinting, while offering high precision, are often expensive and time-consuming, making large-scale production difficult. The requirement for high-quality, biocompatible materials further increases the cost and complexity. We believe that highly utilizing SERS-active plasmonic nanoparticles can enhance the SERS sensitivity. Among various nanoparticle systems, anisotropic gold nanostars (GNS) have attracted significant attention due to their sharp tips, which generate a more intense LSPR effect compared to other shapes of gold nanoparticles.^43,44^ Our laboratory was the first to introduce the use of GNS as a SERS-enhancing platform.^45,46^ However, reported GNS morphologies are still limited for practical SERS applications, primarily due to the polydispersity of morphologies and unclear design principles. Therefore, it is essential to optimize the GNS morphology to achieve highly monodispersed, sharp-branched structures for improved wearable SERS applications.

Herein, we propose a cost-effective, transparent, and flexible wearable SERS sensor. This sensor integrates large, sharply branched GNS, which generate numerous SERS-active “hotspots.” The GNS were deposited on commercially available adhesive Scotch tape at varying concentrations: original, 2 times, 5 times, and 10 times concentrated. The results show that the wearable patch with 10 times more concentrated GNS (WP-4) exhibits maximum SERS enhancements. To demonstrate the practical utility of the wearable SERS sensor, we tested it for the detection of sweat biomarkers including lactate, urea, and glucose in water. The limits of detection (LODs) for lactate, urea, and glucose were 0.7, 0.6, and 0.7 μM, respectively. We further evaluated the performance of our wearable SERS patch (WP-4) during outdoor activities including sitting, walking, and running. The results indicate that we can simultaneously detect and measure the concentrations of lactate, urea, and glucose during these activities.

## Experimental Section

### Materials and Characterization

Ascorbic acid, chloroauric acid (HAuCl_4_), silver nitrate (AgNO_3_, 99.8%), hydrochloric acid (HCl), trisodium citrate (Na_3_C_6_H_5_O_7_), Na-lactate, urea, glucose, and R6G were purchased from Sigma-Aldrich. Milli-Q deionized (DI) water was used throughout the experiment. The STEM images of GNSs were acquired using Aberration Corrected STEM Thermo-Fisher Titan 80–300. UV–vis spectra were recorded using a Shimadzu UV-3600i spectrometer with 1 cm path length cuvettes at room temperature. TEM images were taken using the FEI Tecnai G^2^ Twin TEM system. SEM images were taken using an FEI Verios 460 L.

### Synthesis of Multibranched Sharp-Spiked GNS

Large, sharp-spiked GNS were synthesized using a modified version of a previously reported method.^47^ Briefly, 27 nm gold seeds were first synthesized using an established procedure.^48^ After that, we synthesized multibranched GNS using 27 nm seeds. Briefly, 200 μL of 1 M HCl was added to a solution containing 50 mL of 1 mM HAuCl_4_ and 2 mL of the as-synthesized 27 nm gold seed solution. Subsequently, 2 mL of a 3 mM AgNO_3_ solution and 1 mL of 100 mM ascorbic acid were added to the mixture. The solution was stirred for 2 min before being used to prepare the wearable SERS substrate.

### Preparation of GNS Substrate

The GNS solution was concentrated 10-fold by letting it sit for 48 h. The concentrated GNS solution was then drop-cast onto the adhesive tape and allowed to air-dry for 2 h.

### Raman Measurements

Raman measurements were performed by using a laboratory-built portable Raman instrument having a 785 nm laser source (Rigaku Xantus TM-1 hand-held Raman device), a fiber optic probe (InPhotonics RamanProbe), a spectrometer (Princeton Instruments Acton LS 785), and a CCD camera (Princeton Instruments PIXIS: 100BR_eXcelon). The laser power of the Rigaku Xantus TM-1 was set at 50 mW, and the exposure time was set at 3 s. The wearable patch was initially placed on specific areas, such as the forehead or neck of the volunteers, with the GNS surface in contact with the skin. This allowed the analytes to get in contact with the hot spots of GNS. The patch remained there for a predetermined duration before being removed. Following the removal, SERS measurements were taken. SERS measurements were conducted after removal of the patch from the skin.

## Results and Discussion

### Preparation of Wearable SERS Patches

**Figure 1:**
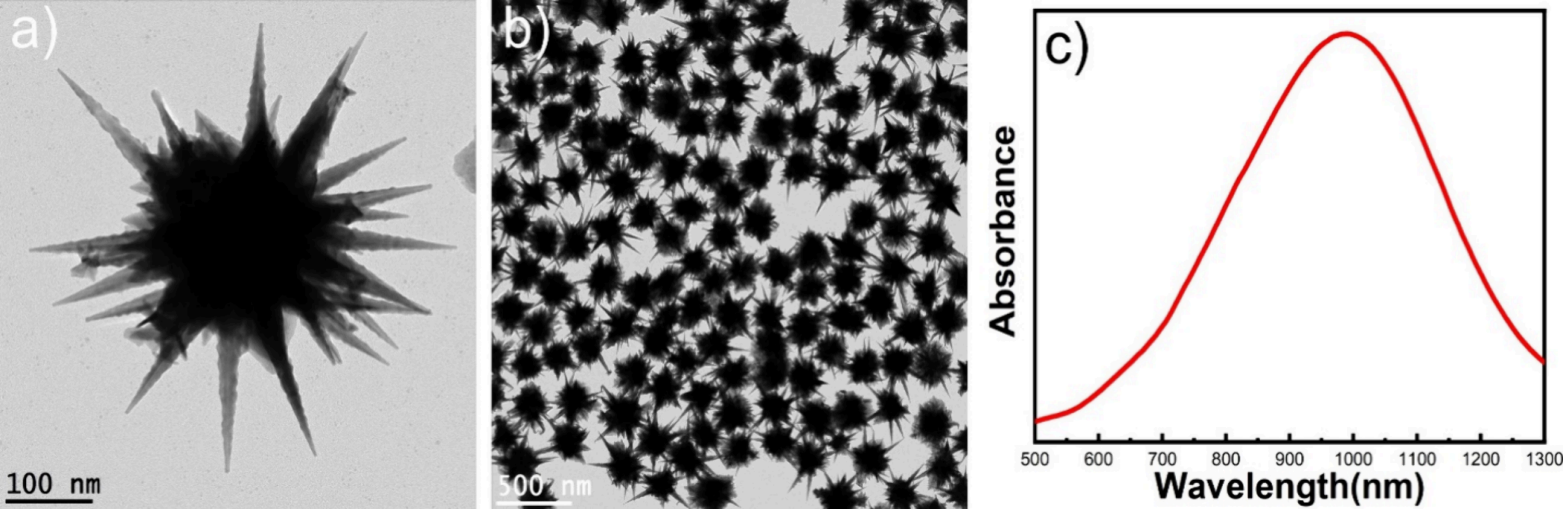
TEM images (a, b) and UV–vis absorbance spectra (c) of multibranched GNS.

In this study, we selected multibranched, large, and sharp-spiked surfactant-free GNS to prepare a wearable SERS patch. The surfactant-free GNS possesses a unique multibranched morphology and LSPR properties, enhancing the SERS performance.^43,49,50^ The morphological tunability of GNS depends on various synthesis parameters including the concentrations of AgNO_3_, ascorbic acid, HAuCl_4_, HCl, and gold seed size.^43,44,51^ Among these parameters, seed size plays an important role in the development of multiple spikes and the overall larger size of the GNS, as it creates multiple nucleation centers for spike growth.^49^ In this study, we selected 27 nm seeds to produce multibranched, large, and sharp-spiked surfactant-free GNS, enhancing the SERS performance of a wearable SERS patch. Figure 1a,b shows transmission electron microscopy (TEM) images of the multibranched GNS, which indicates that the GNS morphology is highly monodispersed. The spike length, measured from the core surface of the GNS, was approximately 200 nm. The UV–vis absorbance spectra revealed that the LSPR peak maximum of the GNS was 985 nm (Figure 1c).

**Figure 2:**
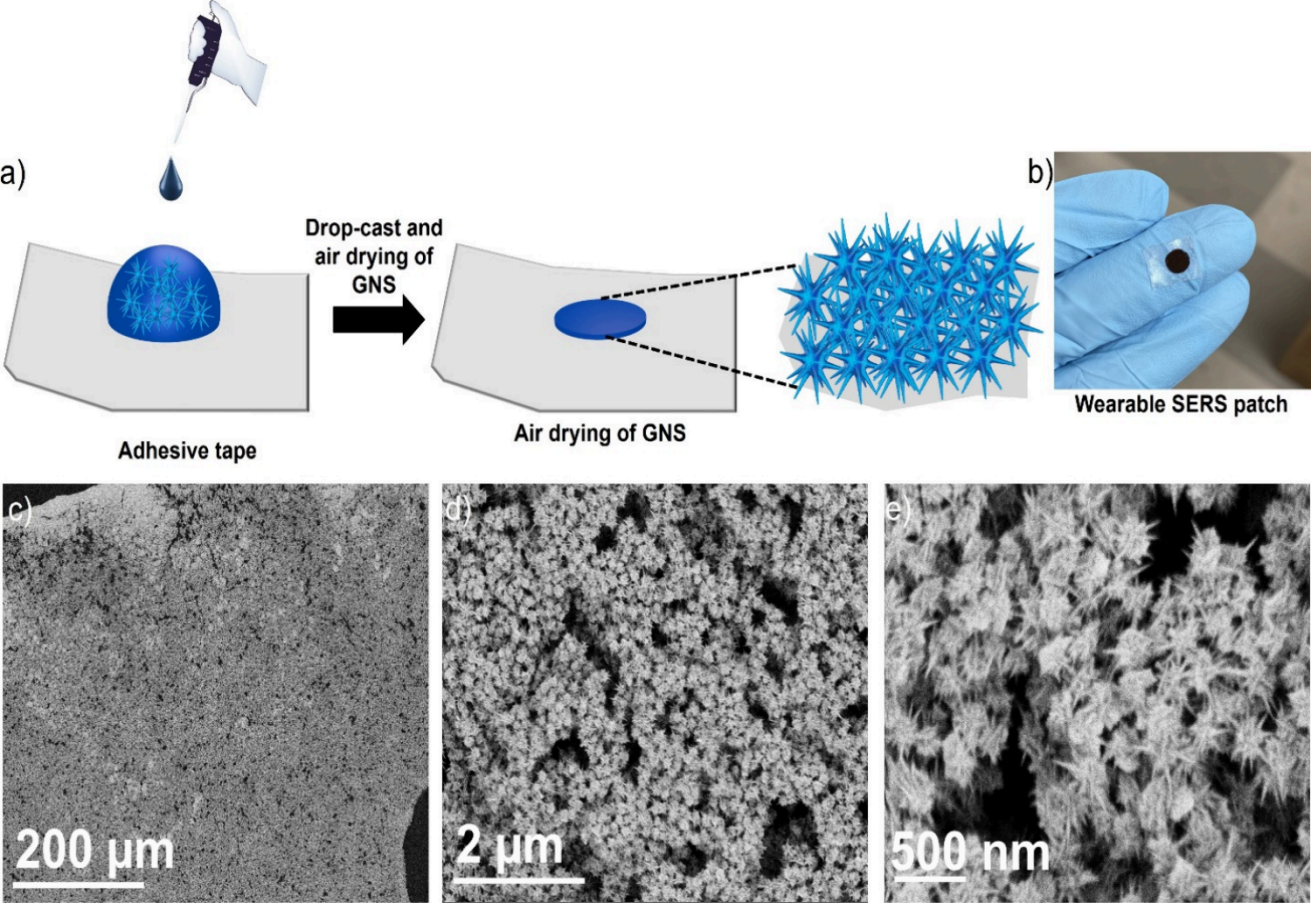
Schematic for the preparation of the wearable patch (a). The photograph of the GNS wearable patch (b). SEM images of the wearable patch at different magnification (c–e).

Figure 2a illustrates the schematic of the multibranched GNS wearable patch preparation, which involves a straightforward method of depositing highly concentrated GNS onto an adhesive tape. The highly hydrophobic nature of the adhesive tape causes the GNS to concentrate in a small area. After depositing the GNS solution, it was air-dried. In this study, we selected four different concentrations of GNS for the preparation of the wearable patch. We concentrated the GNS solution to 2 times, 5 times, and 10 times the original concentration to prepare wearable patches (WP-1, WP-2, WP-3, and WP-4). The patches WP-1, WP-2, WP-3, and WP-4 correspond to the original concentration, 2 times, 5 times, and 10 times concentrated GNS, respectively. The photograph of WP-4 shows that the GNS is concentrated in one spot on the adhesive tape (Figure 2b). Figure S1 exhibits the scanning electron microscopy (SEM) images of WP-1, WP-2, and WP-3. Figure 2c displays the SEM images of WP-4, indicating that the GNS were highly concentrated and uniformly distributed on the adhesive tape. The magnified SEM images revealed that the morphology of the GNS was retained (Figure 2d,e).

### SERS Performance of the Wearable Patches

**Figure 3:**
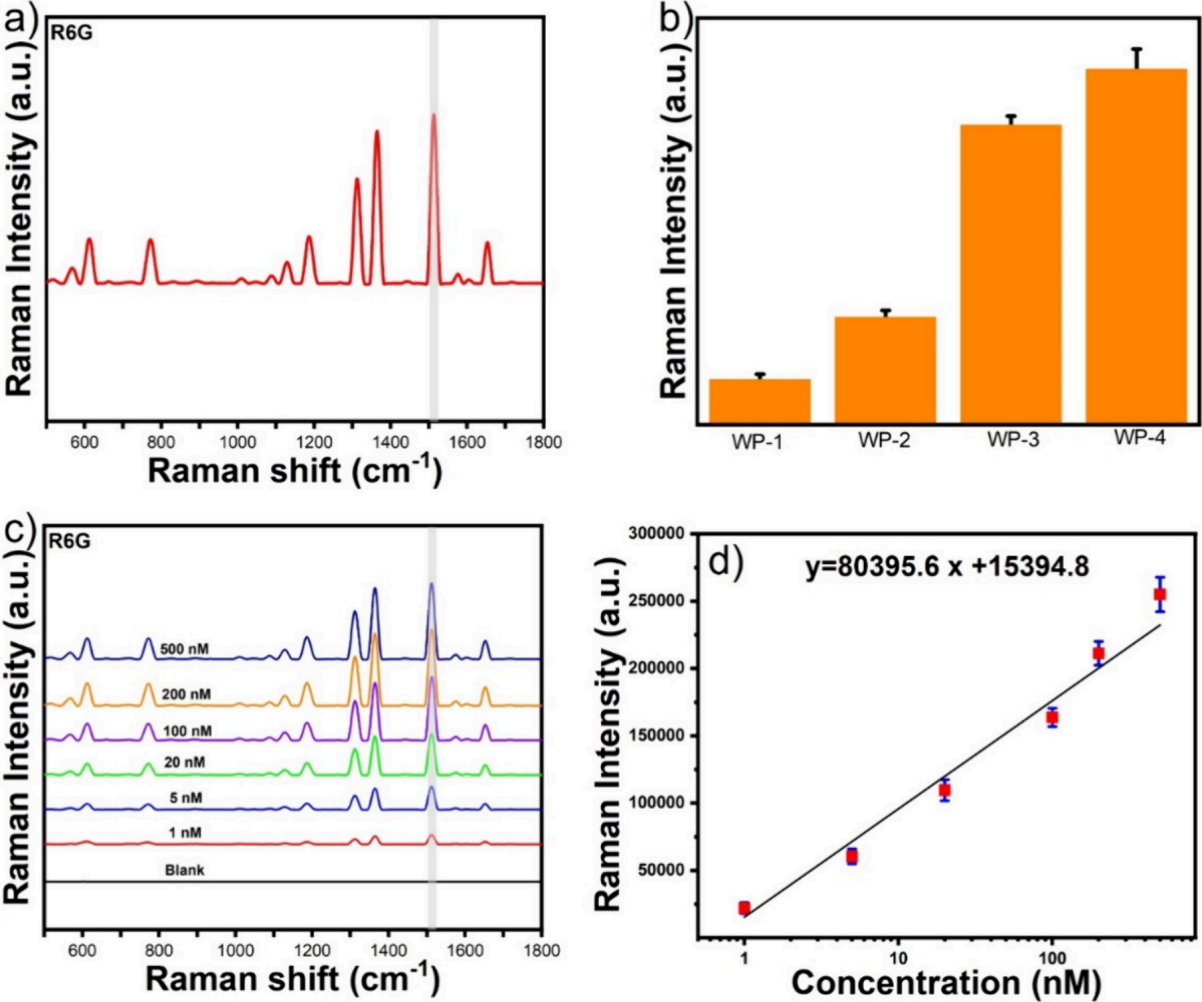
SERS spectra of R6G with WP-4 (a). The SERS peak intensity of WPs indicates that WP-4 has the maximum SERS enhancements (b). The SERS spectra of R6G at different concentrations ranging from 500 to 1 nM (c). The calibration curve of R6G (d).

We investigated the SERS performance of the WPs using a portable Raman instrument. Figure 3a shows the SERS peak intensity of R6G at a concentration of 100 nM with WP-4, highlighting the most intense SERS peak of R6G at 1511 cm^–1^. We selected the 1511 cm^–1^ peak to compare the WPs. Figure 3b displays the SERS peak intensities of R6G at 1511 cm^–1^, indicating that WP-4 provides a much stronger SERS enhancement than that of the other WPs. As expected, the WP-4 substrate exhibited the maximum SERS enhancement, which is probably due to the presence of multiple GNS-generating ultrahigh electric field enhancements.

We further investigated the AEF of the wearable patch. We evaluated the analytical enhancement factor (AEF) of the GNS patch by using the highest SERS signal of R6G at 1511 cm^–1^. The AEF of the GNSs was determined using the AEF formula, as shown in eq 1:
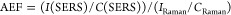
1where *I*_(SERS)_ and *I*_Raman_ are the Raman peak intensities of the characteristic R6G peak at 1511 cm^–1^ in the SERS spectrum and normal Raman spectrum, respectively. The *C*_(SERS)_ and *C*_Raman_ terms are the R6G concentration in the SERS spectrum and the normal Raman spectrum, respectively. The enhancement factor of the WP-1, WP-2, WP-3, and WP-4 patches was calculated to 1.6× 10^4^, 1.4 × 10^5^, 0.8× 10^7^, and 7.9 × 10^8^, indicating that WP-4 is a highly efficient SERS substrate than the other WPs.

Figure 3c shows the SERS spectra of R6G at different concentrations from 500 to 1 nM using WP-4. The calibration curve (Figure 3d) between the concentration of R6G and the Raman intensity at 1511 cm^–1^ displayed a linear correlation between SERS peak intensity of R6G at 1511 cm^–1^ and the logarithmic concentration of R6G. The LOD of R6G was calculated to be 0.01 nM, with a good signal-to-noise ratio (S/N = 3.5), indicating that ultrahigh sensitivity was achieved with WP-4.

### SERS Detection of Lactate, Urea, and Glucose

Sweat offers valuable preliminary insights into nutritional and metabolic status.^4,52^ It contains various important biomarkers that can aid in risk assessment, diagnosis, and monitoring of treatment responses. Among these, lactate, urea, and glucose are particularly significant. For instance, lactate is an important biomarker in sweat for detecting fatigue and inadequate oxidative metabolism. During intense physical activity, elite athletes may experience local lactate buildup in their muscles, which can lead to pain, fatigue, and soreness. Lactate concentrations in the human body typically range from 0 to 25 mM.^53−55^ Similarly, urea, a metabolic byproduct and an important biomarker for kidney function, has a concentration in sweat of approximately 0 to 20 mM in healthy individuals, with elevated levels indicating an increased risk of heart failure.^56^ Glucose concentration in body fluids is another crucial marker for evaluating glucose metabolism disorders. In healthy individuals, sweat glucose levels range from 0.02 to 0.6 mM.^57^ High blood glucose levels, typically associated with diabetes, increase the risk of morbidity and mortality.^6^ Therefore, an effective wearable patch that simultaneously detects glucose, urea, and lactate in sweat is highly desirable, offering comprehensive insights into the human physiological status and metabolism.

**Figure 4:**
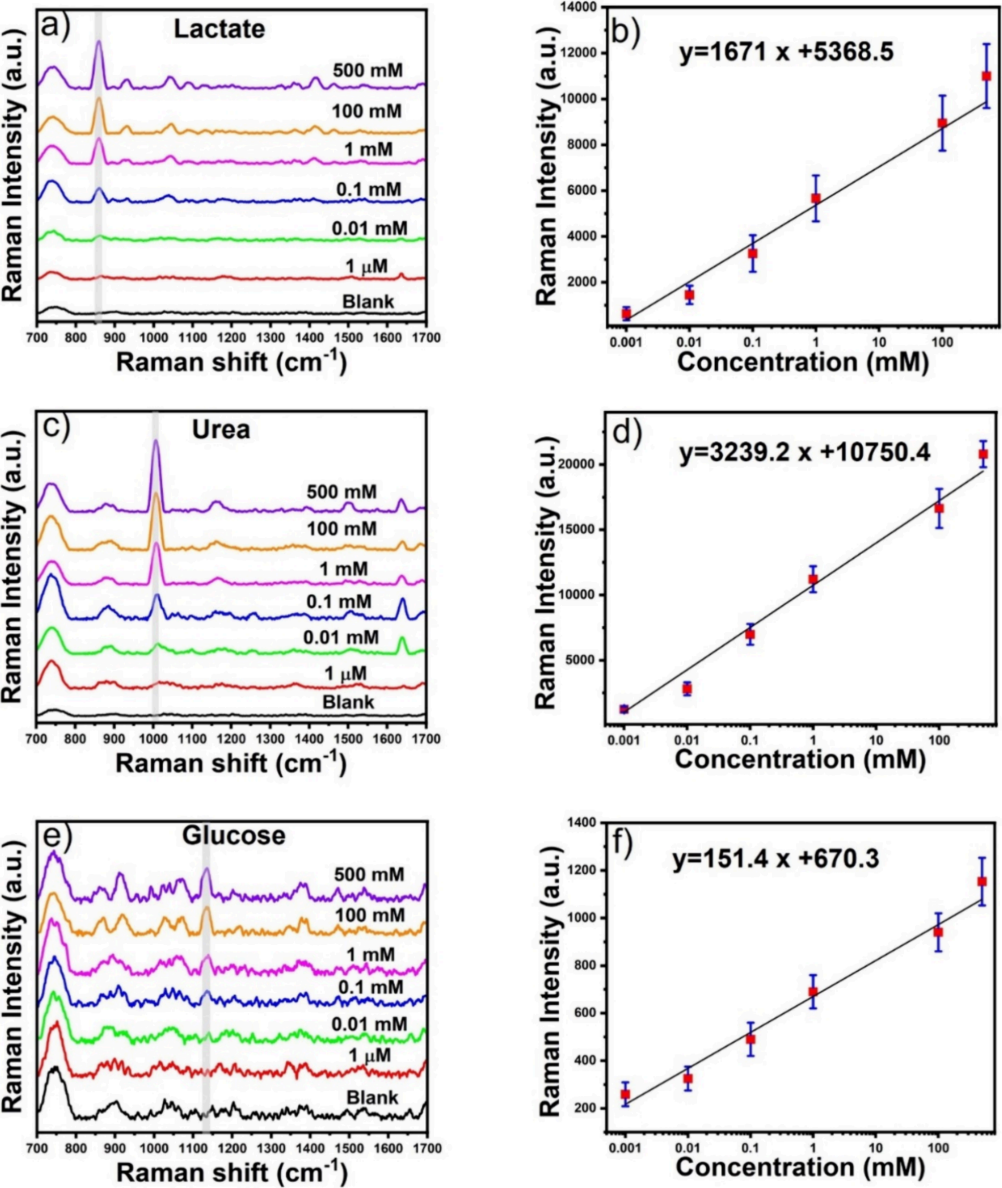
SERS spectra and the corresponding calibration curve of lactate at different concentrations ranged from 500 mM to 1 μM (a, b). The SERS spectra and the corresponding calibration curve of urea at different concentrations ranging from 500 mM to 1 μM (c, d). The SERS spectra and the corresponding calibration curve of glucose at different concentrations ranging from 500 mM to 1 μM (e, f).

To demonstrate the practicality of our sensor for wearable SERS analysis, we selected lactate, urea, and glucose in sweat as the analytes. Quantitative detection of these biomarkers provides valuable information about several diseases. We used WP-4 for further SERS detection of the three sweat biomarkers: lactate, urea, and glucose. Figure 4a–f shows the SERS spectra and corresponding calibration curves of lactate, urea, and glucose. The most intense SERS peaks for lactate, urea, and glucose appear at 863, 1008, and 1140 cm^–1^, respectively. These results are consistent with previously reported findings.^58−60^ Moreover, Figure S2 shows the SERS spectra of glucose and urea at 500 mM concentration, which indicates that the characteristic SERS peak of glucose at 1140 cm^–1^ is distinct and does not interfere with the SERS peak of urea, which appears at 1163 cm^–1^. The calibration curve of lactate, urea, and glucose shows a linear correlation between the logarithm concentration of the analytes and the Raman intensity of the analytes at maximum SERS signal intensity. It is important to note that the most intense SERS peaks for lactate, urea, and glucose do not overlap with each other. Therefore, our SERS sensor exhibited high specificity for detecting lactate, urea, and glucose, even when the concentration of these analytes was in the micromolar range. This indicates that the SERS technique is useful for multiplex detection, as the SERS peaks do not overlap with each other. We determined the LOD of the analytes according to the IUPAC definition: LOD = 3.3σ/*S*, where σ and *S* represent the standard deviation of the blank measurements and the slope of the linear equation, respectively. The LOD for lactate, urea, and glucose were achieved at 0.7, 0.6, and 0.7 μM, respectively. It is important to note that our wearable SERS patch exhibits LODs that are significantly lower than the clinically relevant concentrations of lactate, urea, and glucose in sweat.^56^

We have also conducted SERS measurements of the analytes (lactate, urea, and glucose) in real-world environments, including the presence of sodium and potassium salts. Specifically, the SERS measurements of the analytes were carried out with NaCl and KCl at a concentration of 10 mM, which aligns with the typical levels of sodium and potassium ions found in the human body.^61^ Interestingly, the SERS spectra show that the SERS intensities of the most intense peak for lactate, urea, and glucose appearing at 863, 1008, and 1140 cm^–1^, respectively, were not affected by interfering salts such as NaCl and KCl (Figure S3). This study highlights the advantages of the SERS technique, demonstrating that no additional purification is required for the detection of analytes, and enabling simultaneous detection of multiple analytes.

### Real-Time Monitoring of Lactate, Urea, and Glucose Using WP-4

**Figure 5:**
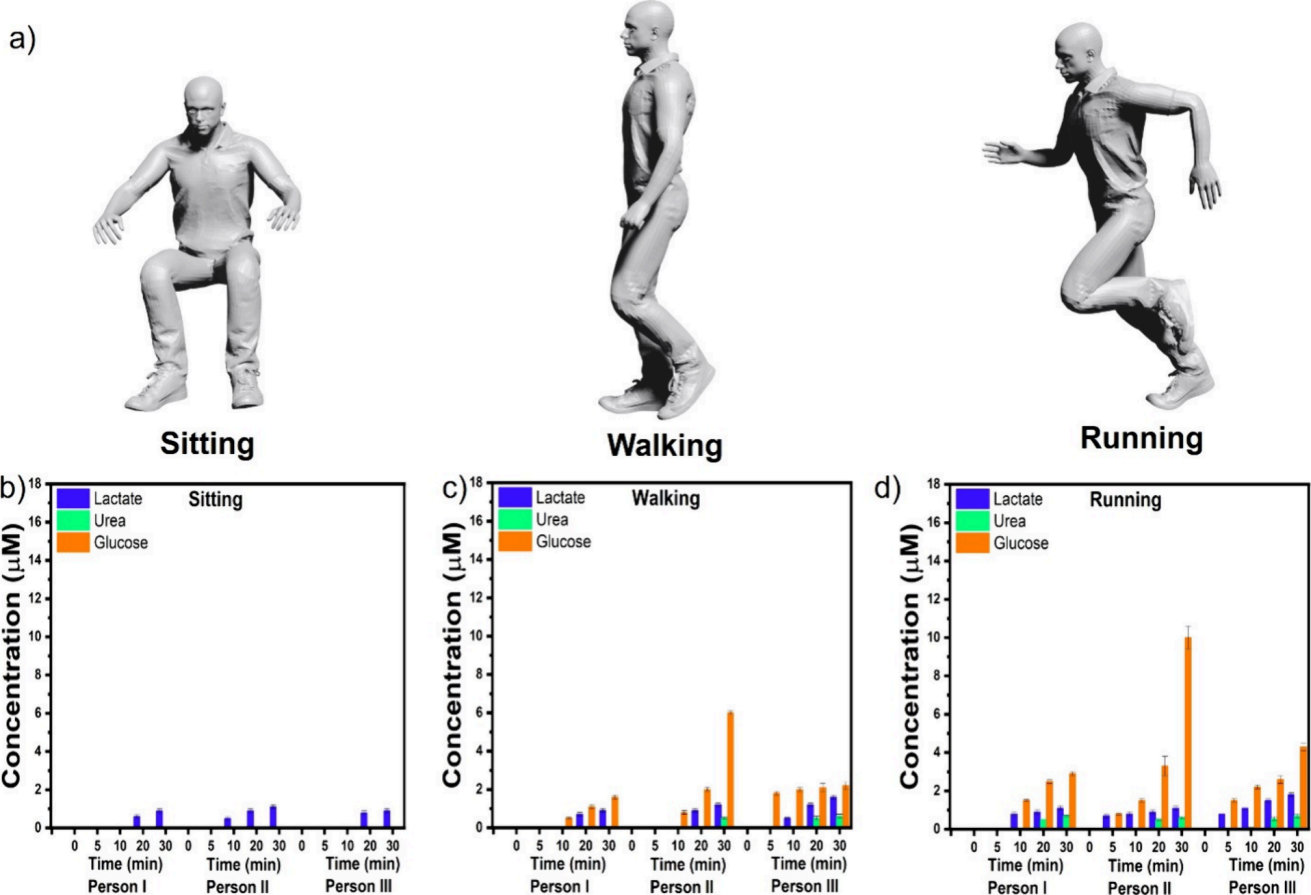
3D model of the sweat monitoring assay for sitting, walking, and running (a). Lactate, urea, and glucose levels for the sitting, walking, and running assays across three volunteers (b–d).

We further investigated the real-time monitoring of the three sweat biomarkers: lactate, urea, and glucose using WP-4. The WP-4 was applied to the foreheads of three healthy individuals at intervals over 30 min while they were sitting, walking, and running in conditions of 28–33 °C and approximately 65% humidity (Figure 5a). Figure S4 shows a photograph of the WP-4 patch applied to the foreheads of three healthy individuals. Figure S5 shows the SERS spectra for the physiological states of sitting, walking, and running. We used the SERS intensity of the characteristic peaks at 863, 1008, and 1140 cm^–1^, respectively, for the quantitative analysis of lactate, urea, and glucose, respectively. The concentrations of the analytes were determined using the calibration equations for lactate, urea, and glucose discussed earlier. Notably, we observed a micromolar concentration of lactate after 20 min of sitting (Figure 5b). However, there were no significant SERS signals for urea and glucose during the sitting experiment, indicating that more time is needed to detect SERS signals for these biomarkers while sitting. Notably, distinct SERS signals for lactate, urea, and glucose appeared during the 20–30 min of walking (Figure 5c), indicating increased sweat secretion. During running, the concentrations of lactate, urea, and glucose in sweat increased more rapidly than those during walking and sitting (Figure 5d). Overall, this experiment shows that our wearable SERS sensor can be useful for monitoring sweat biomarkers in point-of-care settings.

By utilizing highly concentrated multibranched GNS on adhesive tape for the sensitive detection of three important biomarkers: lactate, urea, and glucose in sweat, we achieved sensitivities of up to 0.7, 0.6, and 0.7 μM for lactate, urea, and glucose, respectively. These values are significantly below the clinically relevant concentrations for these biomarkers in health-risk patients.^56^ It is important to note that the amount of sweat produced depends on factors like environmental temperature and humidity, as well as physiological conditions such as age and body weight.^54^ We postulate that the low concentrations observed in our study were influenced by these factors. We believe that the low concentrations observed in our study were influenced by these factors. However, it is beyond the scope of our current study to investigate these parameters and will be addressed in future research. For potential applications, the proposed wearable SERS patch for rapid detection of multiple small molecules such as lactate, urea, and glucose can assist emergency physicians in quickly diagnosing multiple disorders or diseases. Additionally, point-of-care sweat biomarker testing could serve as an alternative to routine primary care visits, allowing for early disease detection in patients with multiple risk factors. Ultimately, our simple and rapid SERS wearable patches could be especially valuable for global health applications in remote or resource-limited settings, where access to advanced laboratory facilities is limited.

## Conclusions

In conclusion, we have presented a simple, sensitive, and cost-effective approach to fabricating commercially available adhesive tape with ultrahigh plasmonically active large multibranched GNS. We optimized the SERS sensitivity of the wearable patch by increasing the concentration of GNS on the adhesive tape. The results show that the wearable patch (WP-4) with a high concentration of GNS (10 times higher than the original GNS synthesis concentration) exhibits maximum SERS enhancement compared to other patches. We utilized this highly sensitive WP-4 for the direct detection of sweat biomarkers: lactate, urea, and glucose. The LODs achieved were 0.7, 0.6, and 0.7 μM, respectively, which are significantly lower than the clinically relevant concentrations of these biomarkers in sweat. We further evaluated the performance of our wearable SERS patch during outdoor activities, including sitting, walking, and running, without any sample preparation or amplification techniques. The results show that our wearable SERS patch can successfully detect the analytes under a wide range of physical conditions. Overall, our SERS wearable patch provides a promising pathway to accelerate the development of low-cost, label-free, wearable SERS sensors for home-care diagnostics.

## Supporting Information Available

SEM images of wearable patches; SERS spectra of glucose and urea demonstrating distinct SERS peak of glucose and urea; SERS spectra of lactate, urea, and in the presence of NaCl and KCl; photograph of the wearable patch on a volunteer’s forehead; and SERS spectra of the patches after sitting, walking, and running of three volunteers with time (PDF)
