# Supplementary material for: A Simple and Sensitive Wearable SERS Sensor Utilizing Plasmonic-Active Gold Nanostars
Source: ACS Omega. 2024 Sep 5;9(37):38897–905. doi: 10.1021/acsomega.4c05140 (PMC11411535; doi:10.1021/acsomega.4c05140)
Supplement: Supplementary file 1 — ao4c05140_si_001.pdf [file ao4c05140_si_001.pdf]

Supporting Information

A Simple and Sensitive Wearable SERS Sensor Utilizing Plasmonic-Active Gold Nanostars

Supriya Atta, [a, b] *Yuanhao Zhao,* [a, b] *Sebastian Sanchez*[c]*, Tuan Vo-Dinh* [a, b, c(*)]

[a] Fitzpatrick Institute for Photonics, [b] Department of Biomedical Engineering, [c] Department of Chemistry, Duke University, Durham, NC 27708, USA

(*) Corresponding Author: tuan.vodinh@duke.edu

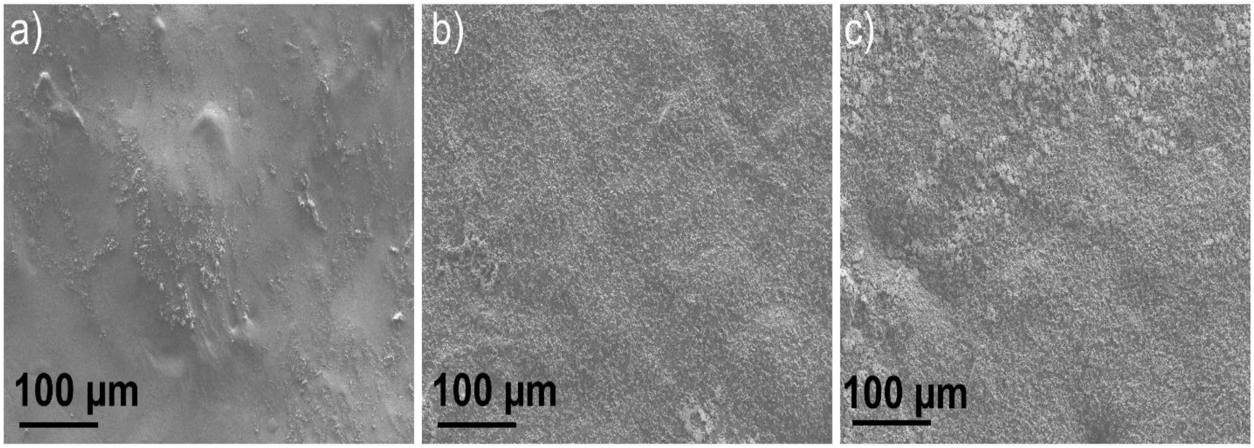

Figure S1. SEM images of WP-1, WP-2, and WP-3 (a-c).

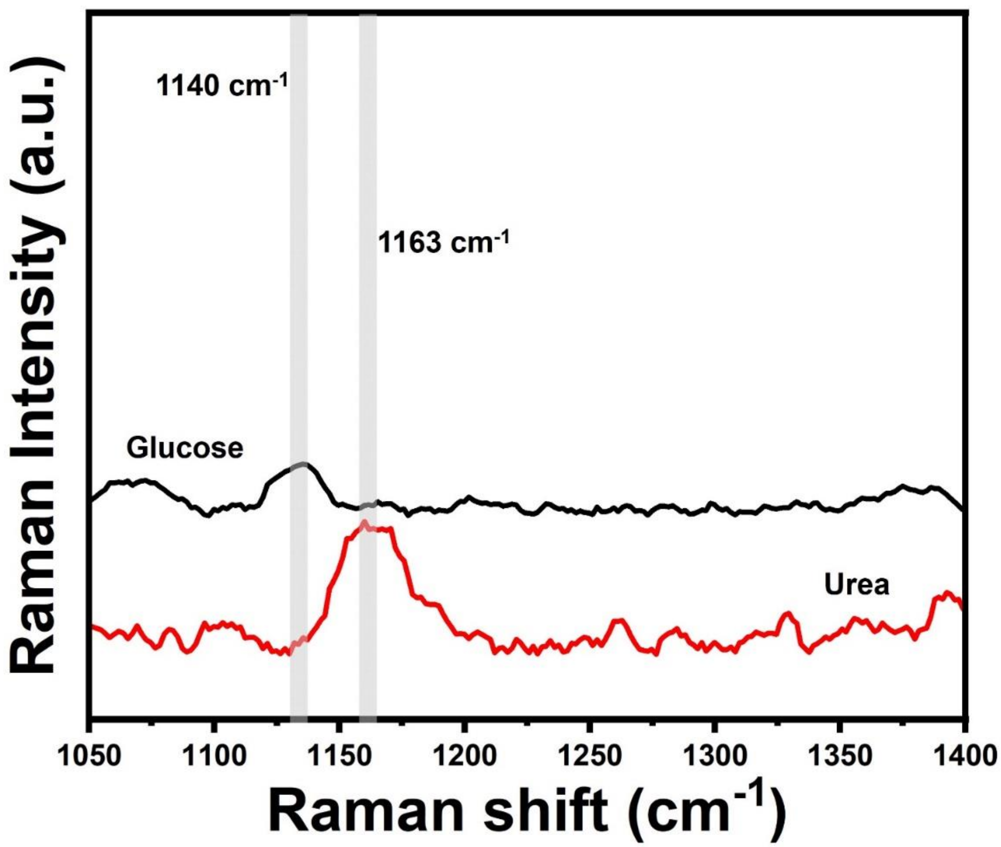

Figure S2. The SERS spectra of glucose and urea demonstrating that the characteristic SERS peak of glucose at 1140 cm^{-1} is distinct and does not interfere with the SERS peak of urea, which appears at 1063 cm^{-1}.

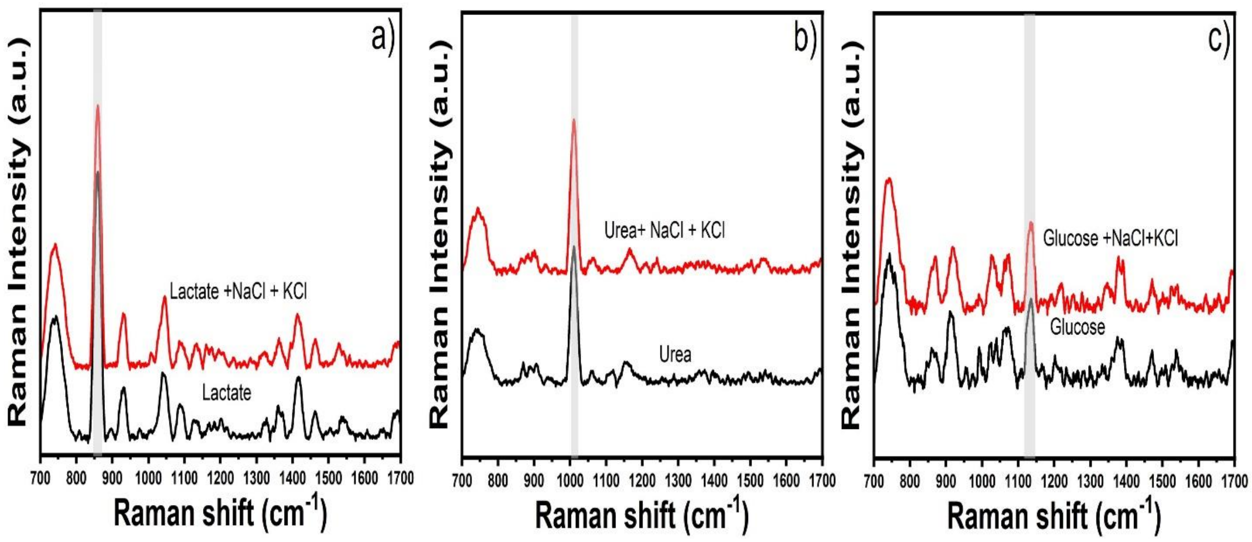

Figure S3. The SERS spectra of lactate, urea, and glucose are shown with (red) and without (black) NaCl and KCl (a-c).

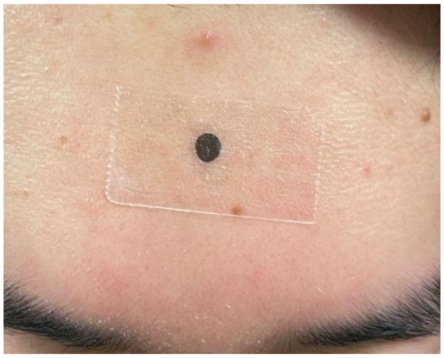

Figure S4. Photograph of the wearable patch on a volunteer's forehead.

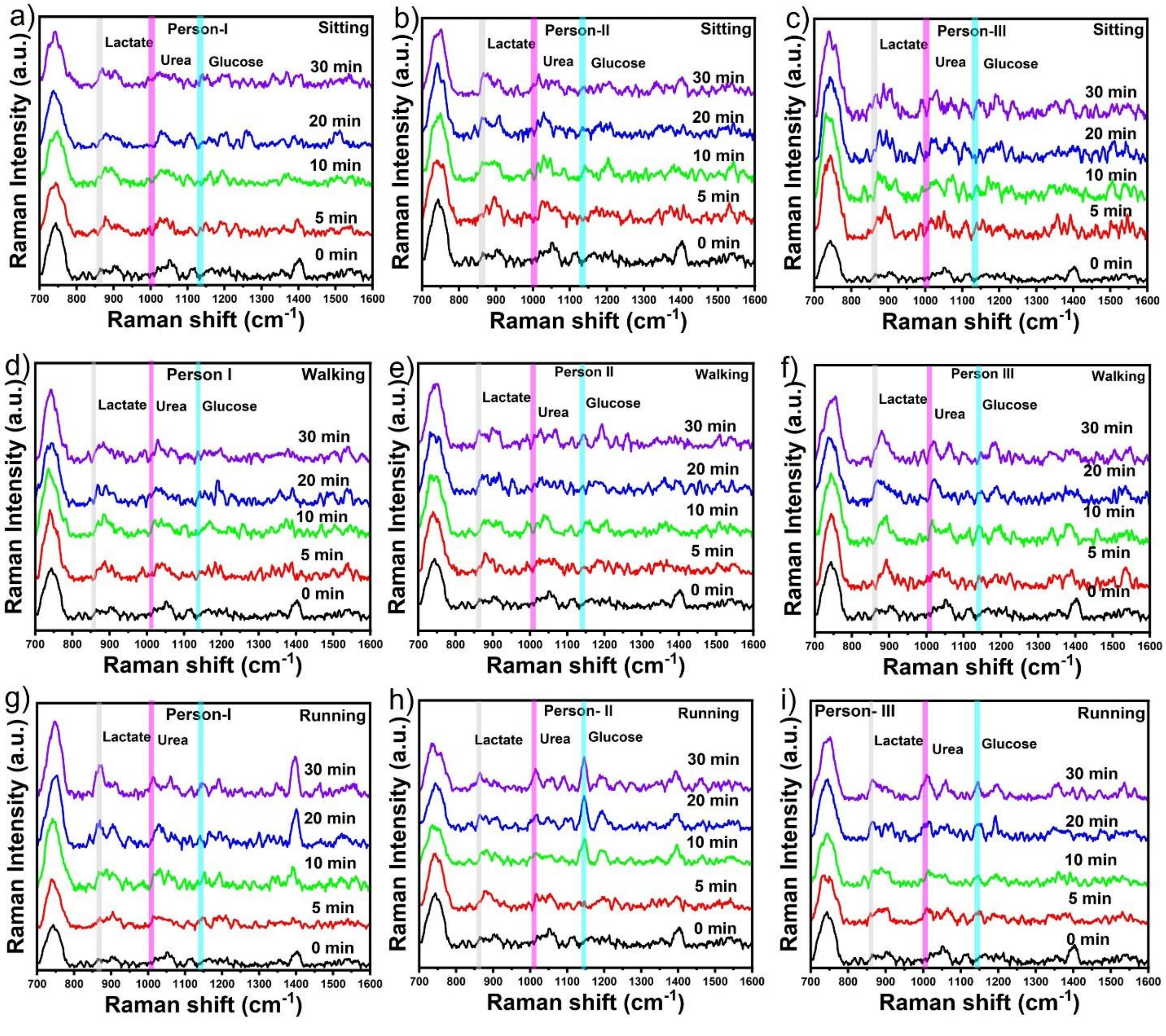

Figure S5. The SERS spectra of the patches after sitting (a-c), walking (d-f), and running (g-i) of three volunteers with time.
